# Extraction of 3,4,4′-Trichlorocarbanilide from Rat Fecal Samples for Determination by High Pressure Liquid Chromatography–Tandem Mass Spectrometry

**DOI:** 10.3390/ijerph120708125

**Published:** 2015-07-15

**Authors:** Rebekah C. Kennedy, Russell R. Fling, Paul D. Terry, Fu-Min Menn, Jiangang Chen, Christopher J. Borman

**Affiliations:** 1 Comparative and Experimental Medicine, University of Tennessee, Knoxville, TN 37996, USA; E-Mail: rkenne12@vols.utk.edu; 2 Department of Public Health, University of Tennessee, Knoxville, TN 37996, USA; E-Mails: pdterry@utk.edu (P.D.T.); jchen38@utk.edu (J.C.); 3 Department of Microbiology, University of Tennessee, Knoxville, TN 37996, USA; E-Mail: rfling@vols.utk.edu; 4 Department of Surgery, University of Tennessee Medical Center, Knoxville, TN 37920, USA; 5 Center for Environmental Biotechnology, University of Tennessee, Knoxville, TN 37996, USA; E-Mail: fmenn@utk.edu; 6 Department of Natural Sciences, Heritage University, Toppenish, WA 98948, USA

**Keywords:** triclocarban, antimicrobials, feces, HPLC-MS/MS

## Abstract

Triclocarban (3,4,4′-Trichlorocarbanilide; TCC) in the environment has been well documented. Methods have been developed to monitor TCC levels from various matrices including water, sediment, biosolids, plants, blood and urine; however, no method has been developed to document the concentration of TCC in fecal content after oral exposure in animal studies. In the present study, we developed and validated a method that uses liquid extraction coupled with HPLC-MS/MS determination to measure TCC in feces. The limit of detection and limit of quantitation in control rats without TCC exposure was 69.0 ng/g and 92.9 ng/g of feces, respectively. The base levels of TCC in feces were lower than LOD. At 12 days of treatment, the fecal TCC concentration increased to 2220 µg/g among 0.2% w/w exposed animals. The concentration in fecal samples decreased over the washout period in 0.2% w/w treated animals to 0.399 µ/g feces after exposure was removed for 28 days. This method required a small amount of sample (0.1 g) with simple sample preparation. Given its sensitivity and efficiency, this method may be useful for monitoring TCC exposure in toxicological studies of animals.

## 1. Introduction

Triclocarban (3,4,4′-trichlorocarbanilide; TCC) is a chlorinated urea commonly used as a broad range antimicrobial in personal care products [1]. Human exposure to TCC primarily occurs through dermal absorption with the use of TCC-containing bar soaps [2], where approximately 0.6% of the applied amount is absorbed through the skin [2]. Biomonitoring investigations have detected TCC in several environmental and biological matrices [2,3,4,5]. Given its widespread use, interest in the health impact of TCC to the general public has increased in both academic and regulatory communities. The FDA recently presented a proposed ruling holding manufacturers of nonprescription antimicrobials, including TCC, responsible to prove the safety and efficacy of these compounds over regular soap [6]. This proposed ruling comes amidst concerns of possible antimicrobial resistance and endocrine disruption activities during routine use in humans [6,7,8,9].

After topical application, the bulk of TCC enters the wastewater treatment process where current treatment technologies can only transfer up to 79% of TCC to waste water sludge [1]. With high octanol-water and organic carbon partition coefficients, TCC has a high propensity to sorb to the hydrophobic components of sludge and soil (log *K*_ow_ = 4.9 and *K*_oc_ = 50,118 L/kg, respectively), and is environmentally persistent undergoing little degradation for months [10]. When biosolids (*i.e.*, treated wastewater sludge) are applied to agriculture fields as fertilizer, TCC is then transferred to the terrestrial environment raising safety concerns regarding the potential uptake of TCC into the food chain, allowing for a potential secondary human exposure route [11,12]. Previously, we demonstrated that TCC exposure through the diet, during lactation, lead to TCC concentration in the milk of exposed dams and substantially reduced rat offspring survival [13]. These results highlight the importance of monitoring TCC levels in various biological matrices to investigate and prevent potential health consequences [13].

Methods of TCC detection have been reported in both solid and liquid biological matrices (urine, blood, and fingernails) [14]. Oral TCC exposure in animals has been used to investigate its potential endocrine-disrupting properties and reproductive toxicity [7,9,15,16,17,18,19]. However, to our knowledge, no analytical method to monitor TCC concentration in a semi-solid fecal matrix has been published. In this study, simple liquid extraction was applied followed by HPLC-MS/MS determination to estimate TCC concentration in fecal samples collected from an animal feeding study. The application of this method may facilitate the assessment of TCC exposure in biological matrices.

## 2. Experimental Methods

### 2.1. Chemicals and Reagents 

Acetone (99.5% purity), acetonitrile (99.9% purity), methanol (99.9% purity) and water (purity grade: Optima™) were purchased from Thermo Fisher Scientific (Waltham, MA, USA). TCC (99% purity), carbon-13 labeled TCC (^13^C_6_-TCC, quantitation reference) and ^13^C_6_-2,4,5-trichlorophenoxyacetic acid (TCPAA, 99% purity, internal standard) were purchased from Cambridge Isotope (Tewksbury, MA, USA) and prepared in methanol. Stock solutions of TCC (5 mg/mL) were prepared in acetone and TCC standards (0–500 ng/mL, or 0–500 ppb) were prepared from stock solution in methanol. TCC standards, ^13^C-TCC and TCPAA were stored at −20 °C until use.

### 2.2. Animal Fecal Samples Collection and Preparation

Feces were collected from female Sprague Dawley rats (Harlan Laboratory, Dublin, VA, USA). Briefly, rats (n = 4 per group) were weight ranked and randomized to control or TCC treatment groups and fed Harlan chow diet (2020X) or 2020X supplemented with 0.2% w/w TCC *ad libitum* for 4 weeks beginning at the post-natal day (PND) 22 followed by 4 weeks washout with 2020X only. Feces were collected prior to treatment initiation at PND 22 and after 12 days of treatment. Fecal samples were also collected throughout the washout period at 2, 8, and 28 days after the withdrawal of TCC exposure. Samples were snap frozen and stored at −80 °C until analysis. The Animal Use and Care Committee at University of Tennessee, Knoxville, approved all research protocols used in this report. The studies were conducted in a facility fully accredited by the Association for Assessment and Accreditation of Laboratory Animal Care.

### 2.3. Fecal Sample Extraction and Preparation

To extract TCC from fecal samples, 50 µL of 500 ng/mL ^13^C_6_-TCC was added to 0.1 g thawed feces and thoroughly mixed with a countertop vortex at maximum speed for 1 minute. Next, 5 mL of 80:20 acetonitrile/H_2_O was added to each sample and vortexed at maximum speed for 30 s. After vortexing, samples were sonicated for 30 min and centrifuged at 1500 rpm for 8 min at 21 °C followed by 0.45 µm filtration prior to blow down under nitrogen flow to 1 mL. Samples were reconstituted to 2 mL with a 1:1 mixture of methanol/H_2_O. A 300 µL aliquot of the mixture plus 6 µL of 2500 ng/mL ^13^C_6_ TCPAA was added to auto-sampler vials prior to analysis.

### 2.4. HPLC-MS/MS Determination 

Instrumental protocols followed were from EPA Method 1694 [20] and as follows: Quantitation of TCC in the sample extracts was performed on a Dionex™ UltiMate™ 3000 HPLC/TSQ Quantum™ Access Max triple quadrupole mass spectrometer (Thermo Scientific). Chromatographic separation from interferences was performed by injection of 6 μL onto a Thermo Scientific™ Hypersil™ GOLD PFP, 2.1 × 100 mm, 1.9 µm column. The HPLC-MS/MS was run in the ESI negative, MRM (Multiple Reaction Monitoring) mode and quantitation was performed by recording the chromatographic peak area of the coincident precursor and product ions, *m/z*: 312.72 and 160.00, respectively [2]. Instrument conditions of both LC and MS are as follows: The HPLC column compartment was held at 38 °C and the autosampler tray temperature was set at 5 °C. The solvent system consisted of H_2_O with 0.02% acetic acid (mobile phase A) and methanol (mobile phase B). The analyte was separated using a gradient program starting with Time (minute) = 0, A = 40%, B = 60% at 0.3 mL/min; Time = 3, A = 2%, B = 98% at 0.3 mL/min; Time = 5.5, A = 2%, B = 98% at 0.3 mL/min; Time = 5.6, A = 2%, B = 98% at 0.35 mL/min; Time = 12, A = 2%, B = 98% at 0.35 mL/min; Time = 12.05, A = 40%, B = 60% at 0.35 mL/min; Time = 18.5, A = 40%, B = 60% at 0.35 mL/min and Time = 18.6, A = 40%, B = 60% at 0.3 mL/min. The MS conditions used in the method were set as follow: negative Electrospray Ionization (ESI); 200 °C for capillary temperature; 425 °C for vaporizer temperature; 20 (Arb) for sheath gas pressure; 2 (Arb) for Aux gas; and 1.5 mTorr for collision gas pressure. Collision energy was set 17 for TCC and ^13^C-TCC, and at 16 for ^13^C-TCPAA. Product ions were monitored at *m/z* 200.700 for ^13^C-TCPAA; *m/z* 160.000 for TCC; and *m/z* 159.700 for ^13^C-TCC. The signal:noise ratio was set at ˃3.

Thermo Xcalibur^©^ (version 2.1) software was utilized to acquire and analyze data. Concentration of TCC in the sample was determined as the peak area ratio of TCC/^13^C-TCC as compared to the calibration curve derived from TCC concentrations: 2.5, 7.5, 37.5, 125, and 500 ng/mL. Quan Browser in Thermo Xcalibur 2.2^®^ was used to set up the calibration curve (2.5, 7.5, 37.5, 125, and 500 ng/mL). A quadratic log-log calibration curve was used for quantitation. Calibrants were weighted by the inverse of the square of their quantity (1/X^2^).

**Figure 1:**
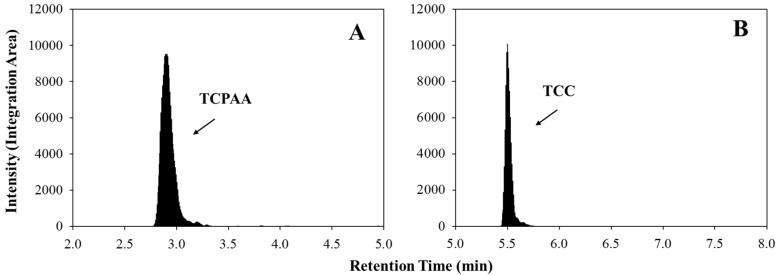
Typical HPLC-MS/MS ion chromatograms from fecal matrix; (**A**) Integration area of representative TCPAA spike in fecal matrix; (**B**) 10 ng/mL TCC spiked blank fecal matrix.

## 3. Results and Conclusions

### 3.1. Method Validation and Quality Control

Typical chromatograms showing integration area of ^13^C-TCPAA, and that of a 10 ng/mL concentration of TCC standard in fecal matrix are shown in Figure 1. The analytical limit of detection (LOD) of the method was 1.46 ng/mL in solvent as defined by the average blank signal plus 3 standard deviations (n = 20). The analytical limit of quantitation (LOQ) was 4.87 ng/mL, which was defined as the average blank signal plus 10 standard deviations. Fecal samples (0.1 g) collected from control dams with no known TCC exposure were spiked with various TCC standards and ^13^C-TCC to characterize the performance of the assay in the presence of matrix. The LOD and the LOQ of the TCC in the fecal matrix were 69.0 and 92.9 ng/g feces, respectively. To determine intra-assay variability, concentration of 10 or 350 ng/mL TCC standard was spiked into control feces (n = 4 per concentration). Day-to-day inter-assay variability was calculated from 4 extractions over a period of 4 days with TCC spiked at both 10 and 350 ng/mL. Percent relative standard deviation (%RSD) of intra-assay variability was 22.4% at 10 ng/mL and 4.99% at 350 ng/mL respectively; the average recovery of TCC was 87.7% at 10 ng/mL and 120% at 350 ng/mL; the inter-assay variability (%RSD) was 30.6% (relative recovery of 73.5%) at 10 ng/mL and 14.1% (relative recovery of 130.6%) at 350 ng/mL over the period of 4 days. The accuracy and precision of the assay in a single extraction day were assessed by repeat analysis of 15 control replicates spiked with either 25 ng/mL or 100 ng/mL TCC (Table 1).

**Table 1:** Assay performance parameters.

Spiked TCC *		Intra-variability		Inter-variability	
		Average (ng/m)L	% RSD **	Average (ng/mL)	%RSD
10 ng/mL	n = 4	8.77	22.4	7.35	30.6
350 ng/mL	n = 4	420	4.99	457	14.1
		Accuracy (%)		Precision (%RSD)	
25 ng/mL	n = 15	98.0		12.8	
100 ng/mL	n = 15	105		16.0	

* Fecal matrix spike of various TCC concentrations; ** Relative standard deviation (RSD).

**Table 2:** Fecal TCC concentration during treatment and washout periods.

Treatment Day	Control	0.2% w/w TCC
0	<LOD	<LOD
12	<LOD	2220 ± 150
Washout Day		
2	<LOD	15.5 ± 3.50
8	<LOD	0.885 ± 0.377
28	<LOD	0.399 ± 0.178

n = 2 animals per group; Concentration shown as µg/g.

### 3.2. Quantification of TCC in Feces Samples 

The assay was applied to determine the concentration of TCC in fecal samples collected from female SD rats during the treatment and a post-TCC exposure washout period. The concentration of TCC in the feces over the study period is shown in Table 2. At PND 22 (pre-exposure), the TCC concentration in fecal samples collected from both control and 0.2% w/w treated animals was below the LOD. At 12 days of treatment, the fecal TCC concentration in control animals was still below the LOD, but increased to 2220 µg/g among 0.2% w/w exposed animals. The concentration of TCC in fecal samples decreased over the washout period in 0.2% w/w treated animals. At 8 days of washout, TCC in the feces of control animals (n = 2) was below the LOD (<69.0 ng/g); in contrast, an average of 0.885 µg/g TCC was detected in fecal samples collected from 0.2% w/w TCC treated animals (n = 2) at the same washout date. At 28 days of washout, the concentration of TCC extracted from control animals (n = 2) remained below the LOD, whereas TCC concentration decreased to 0.399 µg/g in rats (n = 2) exposed to TCC 28 days prior, which reflected discontinuation of exposure.

## 4. Conclusions

We developed a HPLC–MS/MS method for TCC detection from the fecal matrix that required a relative small amount of fecal sample (0.1 g) and utilized a liquid extraction method (Acetonitrile/H2O: 80/20). Our method was based on EPA Method 1694, which is optimized for Group 3 Compounds [20]. TCC elutes at 5.5 minutes, thus it is possible that a shorter column (50 mm) could be used and/or column cleaning could start at 6 minutes for 3 min followed by re-equilibration, which potentially could further improve the analytical efficiency and reduce the solvent required for TCC measurement. Our data demonstrate the ability of this method to monitor TCC concentrations from the fecal matrix with reasonable repeatability and inter-/intra-assay variability. This method may prove useful in animal-based toxicological investigations and, ultimately, studies of TCC exposure and human health.
